# Isoimperatorin, a natural furanocoumarin, ameliorates ulcerative colitis by inducing Treg cell generation and promoting mucosal healing

**DOI:** 10.1186/s10020-025-01334-y

**Published:** 2025-08-07

**Authors:** Yulai Fang, Shichen Min, Hongxin Chen, Zhenxing Zhu, Yanan Li, Yiheng Tong, Jingyi Hu, Lei Zhu, Hong Shen

**Affiliations:** 1 Affiliated Hospital of Nanjing University of Chinese Medicine, Nanjing, China; 2 Jiangsu Province Hospital of Chinese Medicine, Nanjing, China

**Keywords:** Isoimperatorin, Ulcerative colitis, Treg cell generation, Epithelial cell migration, Mucosal healing

## Abstract

**Background:**

Ulcerative colitis (UC), an autoimmune disorder characterized by chronic intestinal inflammation, primarily targets the colonic mucosa. Isoimperatorin is a natural furanocoumarin compound with a variety of pharmacological activities. However, its therapeutic potential and underlying mechanisms in UC pathogenesis remain to be elucidated.

**Methods:**

Mice were administered 2.5% dextran sulfate sodium (DSS) *ad libitum* to construct a colitis model, and isoimperatorin was given by gavage to evaluate its efficacy. Flow cytometry and qPCR were employed to assess the effect of isoimperatorin on Treg cell generation. Tissue immunofluorescence and Western blot were used to investigate the effects of isoimperatorin on the repair of damaged intestinal barrier. Cell scratching and migration were used to examine the effects of isoimperatorin on wound healing and cell migration. Finally, a Treg cell depletion assay was implemented to verify the Treg cell-dependent effect of isoimperatorin on repairing intestinal barrier injury to ameliorate UC.

**Results:**

Oral gavage administration of isoimperatorin (20, 40 mg/kg) significantly improved disease symptoms in UC mice. Isoimperatorin treatment elevated the percentage of Treg cells but had no significant effect on the proportion of Th17 cells in mesenteric lymph node tissues. Isoimperatorin upregulated the expression levels of factors related to mucosal healing and upregulated the expression levels of proteins related to the integrity of the intestinal epithelial barrier. Additionally, isoimperatorin (1, 3 µM) accelerated the migration of colon epithelial cells to facilitate wound healing and also induced the generation of Treg cells in vitro. Finally, Treg cell depletion markedly attenuated isoimperatorin’s therapeutic efficacy in intestinal barrier repair and UC amelioration, indicating a Treg cell-dependent mechanism of action.

**Conclusions:**

Isoimperatorin promotes intestinal mucosal healing and thus improves UC disease symptoms by inducing Treg cell generation.

## Introduction

Ulcerative colitis (UC) is an autoimmune disease characterized by inflammatory activity primarily targeting the intestinal tract (Zilbauer and Heuschkel 2022). The pathogenesis of UC is still unclear, and it may involve environmental factors, genetic susceptibility, immune dysfunction, and microbial imbalance (Du and Ha 2020). Abnormal intestinal immune response, especially Th17/Treg cell imbalance, is a major driver of intestinal inflammation (Souza and Fiocchi 2016). Clinical investigations demonstrate that UC patients exhibit significantly elevated percentages of Th17 cells in both peripheral circulation and colonic mucosa, concomitant with increased IL-17 A production– cytokines that directly contribute to colonic inflammation severity (Gomez-Bris et al. 2023). Conversely, Treg cells have immunosuppressive activity and secrete anti-inflammatory factors such as IL-10 to block inflammatory cell responses. Experimental studies utilizing Treg-specific knockout models in C57BL/6 mice have revealed that targeted deletion of colonic Treg cells accelerates disease progression in DSS-induced colitis. Adoptive transfer of Treg cells demonstrated significant therapeutic efficacy in Rag1^−/−^ mice with CD4 + T cell-driven colitis, manifesting as attenuated body weight reduction, preserved colon length, and improved histopathological scores (Zhang et al. 2016; Thakur et al. 2016). Notably, the Th17/Treg ratio imbalance has been established as a hallmark immunopathological feature in UC and shows a positive correlation with disease progression. Therefore, up-regulation of Treg cell proportion or down-regulation of Th17 cell proportion is an effective strategy for UC management (Göschl et al. 2019).

Mucosal barrier defects constitute a critical pathophysiological determinant of chronic UC progression, perpetuating the inflammatory cycle through compromised epithelial integrity (Salim and Söderholm 2011). With advances in diagnostic and therapeutic approaches, mucosal healing has become the latest treatment target recommended in UC clinical guidelines. The process of intestinal mucosal healing is a complex coordination between epithelial and other cellular components (Leppkes et al. 2018). Epithelial cells adjacent to the wound migrate to the site of injury to reseal the exposed basement membrane, and the closer to the site of injury, the faster the cells migrate (Seidelin et al. 2013). Studies have shown that accelerated epithelial cell migration promotes mucosal healing, thereby alleviating intestinal inflammation. On the contrary, inhibition of epithelial cell migration can impede mucosal healing and exacerbate intestinal inflammation symptoms (Zhang et al. 2023; Guo et al. 2024). Therefore, intestinal epithelial cell migration is essential for restoring the functional integrity of the epithelial barrier and is a critical step in mucosal healing (Oncel and Basson 2022).

Baizhi (*Angelica dahurica* (Hoffm.) Benth. & Hook.f. ex Franch. & Sav.), a traditional Chinese medicine (TCM) with dual food-medicine status, demonstrates therapeutic effects including dispelling wind and relieving pain, resolving surface and dispersing cold, and eliminating swelling and draining pus. Experimental studies in trinitrobenzene sulfonic acid-induced rat UC models demonstrate that oral administration of Baizhi elevates serum IL-10 levels and significantly ameliorates disease symptoms, indicating its potential to enhance immunosuppressive responses (Zhao et al. 2011). In addition, Baizhi is one of the components of the TCM compound Qingchang Huashi formula, and its compound has the effect of regulating the immune balance and repairing the damage of the intestinal mucosa in UC management. The chemical composition analysis of the formula identified isoimperatorin as the principal bioactive constituent derived from Baizhi (Cheng et al. 2022). This finding suggests that isoimperatorin may be the active component of Baizhi in exerting anti-UC effects. However, whether isoimperatorin has immunomodulatory and pro-mucosal healing effects to improve UC needs to be further explored.

## Materials and methods

### Chemicals and reagents

DSS (Cat. No. 160110) was obtained from MP Biomedicals (MW: 36000–50000, MP Biomedicals, Solon, OH, USA). Isoimperatorin (C_16_H_14_O_4,_ Cat. No. HY-N0286) was purchased from MedChemExpress (Houston, USA). 5-Aminosalicylic acid (Cat. No. 79809, 5-ASA) was acquired from Sigma-Aldrich (St Louis, MO, USA). FITC-anti-CD4 (Cat. No. 2608917), APC-anti-Foxp3 (Cat. No. 2513557), PE-anti-CD25 (Cat. No. 2294070), APC-anti-CD4 (Cat. No.2183537) and PE-anti-IL17A (Cat. No.2261900) were purchased from eBioscience (San Diego, USA). The Fixation/Permeabilization Solution Kit (Cat. No. 554714) was provided by BD Biosciences (San Jose, USA). BFA/Monensin (Cat. No. CS1002) and PMA/Ionomycin (Cat. No. CS1001) were bought from MultiSciences (Hangzhou, China). ZO-1 antibody (Cat. No. 33735) was bought from Santa Cruz Biotechnology (Oregon, USA). Claudin-4 antibody (Cat. No. 53156) was bought from Abcam (Cambridge, MA, USA). Actin antibody (Cat. No. 66009), HRP conjugated goat anti-mouse antibody (Cat. No. 15014), and HRP conjugated goat anti-mouse antibody (Cat. No. 15015) were bought from Proteintech (Wuhan, China); TRIzol reagent (Cat. No. 15596026) was purchased from Invitrogen (Carlsbad, CA, USA). Hieff@ qPCR SYBR green master mix (Cat. No. Q71102) and Hifair@ III 1st Stand cDNA Synthesis Super Mix (Cat. No. R32301) were bought from Vazyme (Nanjing, China). Purified anti-CD25 mouse (PC16, Cat. No. C1194) antibody was bought from Leinco (Chicago, USA).

### Mice

SPF grade male C57BL/6 mice aged 6–8 weeks (weighing 18–22 g) were purchased from SiPeiFu Biotechnology Co., Ltd (Beijing, China). All animal procedures and experiments were assessed and approved by the Animal Ethics Committee of Nanjing University of Chinese Medicine (Approval NO. 2023DW-083-01) in accordance with the U.S. National Institute of Health Guide for Care and Use of Laboratory Animals.

### Establishment and drug administration of colitis in mice

Male C57BL/6 mice received 2.5% DSS *ad libitum* for 7 consecutive days, followed by normal drinking water for an additional 7 days to establish colitis. The mice were randomly allocated into five groups (*n* = 6): normal group, model group, isoimperatorin low-dose group, isoimperatorin high-dose group, and 5-ASA group. Isoimperatorin doses (20 mg/kg and 40 mg/kg) were selected based on previous pharmacological studies (Wijerathne et al. 2017). 5-ASA was used as a positive control at a dose of 100 mg/kg. From days 8–14, respective treatments were administered via oral gavage to corresponding groups, while normal and model groups received equivalent volumes of vehicle solution.

### Deplete Treg cells in mice

Male C57BL/6 mice received 2.5% DSS *ad libitum* for 7 consecutive days, followed by normal drinking water for an additional 7 days to establish colitis. Purified anti-CD25 mouse antibody was administered via tail vein injection on day 7 to deplete Treg cells in mice (Peng et al. 2024). Mice were randomly allocated to five groups (*n* = 6): normal group, model group, isoimperatorin group (40 mg/kg), Treg cell depletion group (200 µg/mouse), and Treg cell depletion combined with isoimperatorin group. From days 8–14, isoimperatorin was administered *via* oral gavage to treatment groups, while normal and model groups received equivalent volumes of vehicle solution.

### Histological and immunohistochemical analyses

Colon tissue and mesenteric lymph node (MLN) tissue from mice were collected and fixed in 4% formaldehyde. Then, the colon tissue or MLN tissue was embedded in paraffin, cut into 5 μm size sections, and stained with H&E or immunofluorescence. These experimental procedures were provided by Servicebio (Wuhan, China).

### Preparation of single cell suspensions in MLNs

Mouse MLN tissues were harvested, washed once with pre-cooled PBS, transferred to 1.5 mL EP tubes, mechanically dissociated using micro-dissection scissors, resuspended adding 800 µL of pre-cooled PBS, and filtered through a 300-mesh sieve. The filtrate was collected and centrifuged at 1800 rpm at 4 °C for 10 min, and the supernatant was discarded. The cell precipitate was washed once with pre-cooled PBS. Finally, the cells were resuspended in complete RPMI-1640 medium to generate single-cell suspensions from MLNs.

### Flow cytometry assay

For Treg cell staining, single-cell suspensions were stained with 0.65 µL each of FITC-anti-CD4 antibody and PE-anti-CD25 antibody, and incubated at room temperature and protected from light for 30 min. Followed by fixation and permeabilization for 6 h. Then, they were stained with 1µL APC-anti-Foxp3 antibody for 1 h.

For Th17 cell staining, single-cell suspensions were quantified and activated using BFA/Monensin and PMA/Ionomycin for 6 h. After activation, cells were stained with 0.5 µL APC-anti-CD4 for 30 min and then incubated with 1 µL PE-anti-IL-17 A at 4 °C for 1 h after permeabilization.

The flow cytometry was conducted on BD FACS Calibur (BD Biosciences, San Jose, USA). All the data were analyzed using Flowjo 10.0.1 software (Flowjo LLC, USA).

### Quantitative real-time PCR

Total RNA was isolated from colon or MLN tissue using TRIzol Extraction Reagent according to the manufacturer’s instructions. The cDNA synthesis was performed using Hifair@ III 1st Stand cDNA Synthesis Super Mix. The Hieff@ qPCR SYBR master mix was used on a Roche Light Cycler 96 system for RT-qPCR analysis. Primers used are listed in Table 1.

**Table 1:** Primers used for quantitative PCR

Gene	Primer sequences (5′-3′)	
	Forward	Reverse
*Tnf-α*	AAGGCCGGGGTGTCCTGGAG	AGGCCAGGTGGGGACAGCTC
*Il-6*	CCACTTCACAAGTCGGAGGCTTA	AGTGCATCATCGTTGTTCATAC
*Il-1β*	GAATGCCACCTTTTGACAGTG	TGGATGCTCTCATCAGGACAG
*Il-10*	CTTACTGACTGGCATGAGGATCA	GCAGCTCTAGGAGCATGTGG
*Foxp3*	ATGAGTTTTTCCCTTATGGGGAC	GCTGGAAGTTGGACACCTCAA
*Il-17a*	TCAGCGTGTCCAAACACTGAG	CGCCAAGGGAGTTAAAGACTT
*Rorγt*	TCCACTACGGGGTTATCACCT	AGTAGGCCACTTACACTGCT
*Occudin*	GGTGAATGGGTCACCGAGGG	AGCAAAATGTCCAGGCTCCC
*Muc2*	TGTGTTTCAGGCTCCATCAC	TGCAGCCATTGTAGGAAATC
*Egf*	TGGTCCTGCTGCTCGTCTTGG	GTCCGCTGCTGCTCACACTTC
*Tff3*	TGTCAGAGTGGACTGTGGCTACC	GTTTGAAGCACCAGGGCACATTTG
*β-actin*	CTCATGAAGATCCTGACCGAG	AGTCTAGAGCAACATAGCACAG

### Western blot assay

Tissue proteins were extracted using RIPA lysis buffer containing protease inhibitors and phosphatase inhibitors cocktails. The denatured proteins were separated by SDS-PAGE. Subsequently, separated proteins were transferred to nitrocellulose filter membranes. Membranes were blocked with 5% non-fat milk for 2 h at room temperature. Then, membranes were incubated with specific primary antibodies against the membrane overnight at 4 °C and appropriate HRP-conjugated secondary antibodies against the membrane at room temperature for 2 h. Protein bands were visualized with an enhanced chemiluminescent reagent using the ChemiDocTM XRS + system (Bio-rad, Hercules, CA, USA).

### Cell viability analysis

Human colon epithelial cells (NCM 460 cells) were seeded in 96-well plates with 1.2 × 10^5^ cells/well, 180 µL per well. After the cells were attached to the wall, 20 µL of different concentrations of isoimperatorin was added to the experimental wells to make the final concentrations of 0.3, 1, 3, 10, 30, and 100 µM, and an equal volume of solvent (DMSO) was added to the control wells. Six parallel wells in each group were incubated at 37 °C and 5% CO2 for 24 h. Cells were incubated with 20 µL CCK-8 reagent for 4 h prior to analysis. Absorbance at 570 nm was measured using a microplate reader. The cell viability was calculated according to the formula: survival rate = (OD value of experimental wells - OD value of blank wells)/(OD value of control wells - OD value of blank wells) × 100%.

### Wound healing assay

NCM 460 cells were seeded in 12-well plates with 1.2 × 10^5^ cells/well. After the cells were adhered to the wall and grew to a seamless monolayer, they were scratched with a 200 µL pipette tip, washed once with PBS, added with serum-free medium containing isoimperatorin (0.3, 1, 3 µM), and photographed under a microscope (0 h baseline). The plates were incubated at 37 °C and 5% CO_2_ for 24 h. The plates were removed, washed once with PBS, and photographed under a microscope (24 h endpoint). The scratch area was calculated using Image J. The 0 h scratch area was recorded as S1, and the scratch area after 24 h of incubation was recorded as S2, and the cell wound closure rate (%) = (S2-S1)/S1 × 100%.

### Cell migration assay

NCM 460 cells (6 × 10^4^ cells/well) were seeded in the upper chamber of the Transwell (0.5 mL/well), and 1.5 mL of medium was added to the lower chamber. After the cells adhere to the wall, the medium in both chambers is replaced with serum-free medium to starve the cells overnight. The lower chamber was replaced with a medium containing isoimperatorin (0.3, 1, 3 µM) and 10% fetal bovine serum, and the culture was continued at 37 °C with 5% CO_2_ for 24 h. The medium was aspirated and washed three times with PBS, and the inner cells of the upper chamber were wiped off with a cotton swab and washed 3 times with PBS. The cells were fixed with 4% paraformaldehyde for 15 min and stained with 0.1% crystal violet for 15–20 min. After PBS washing, migrated cells were imaged under the microscope.

### Induction of Treg cell generation in vitro

Naïve CD4 + T cells were isolated from MLNs of C57BL/6 mice using a CD4 + CD62L + T Cell Isolation Kit and cultured in RPMI 1640 medium containing 10% FBS. For Treg cell generation, the stimulation conditions were CD3 (3 µg/mL), CD28 (2 µg/mL), TGF-β (3 ng/mL), and IL-2 (100 U/mL). Different concentrations of isoimperatorin (0.3, 1, 3 µM) were also added and the cells were continued to be cultured for 72 h.

### Satistical analysis

All data were presented as means ± S.E.M. and analyzed using GraphPad Prism 8 software. The differences in data between each group were analyzed using one-way ANOVA and Tukey’s test, with *P* < 0.05 indicating significant differences.

## Results

### Isoimperatorin treatment promotes recovery of DSS-induced colitis in mice

**Fig. 1:**
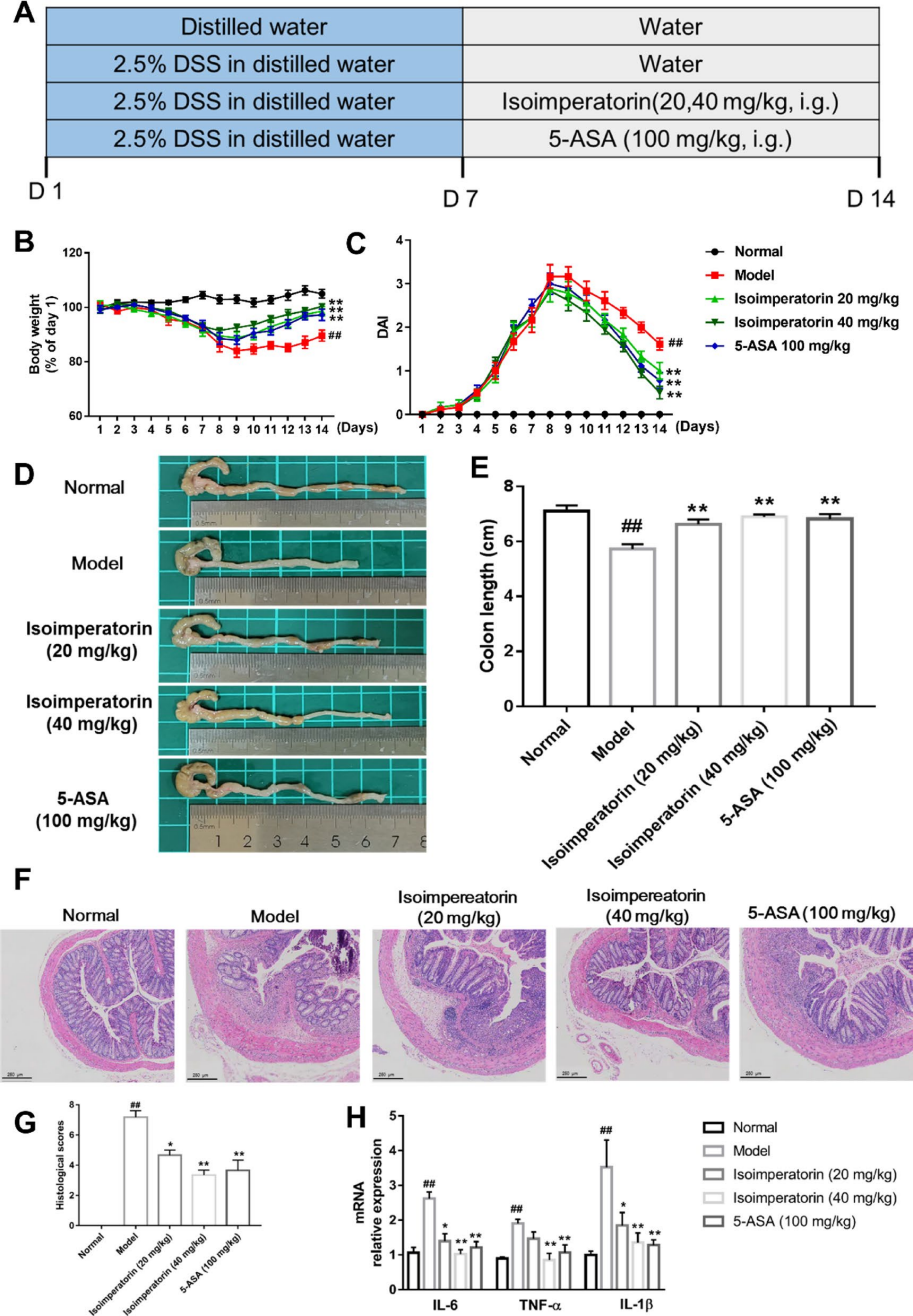
Isoimperatorin treatment promotes recovery of DSS-induced colitis in mice. Colitis was induced in male C57BL/6 mice by drinking with 2.5% DSS for 7 days, followed by normal drinking water for 7 days. Isoimperatorin (20, 40 mg/kg) was orally administered for consecutive 7 days. 5-ASA (100 mg/kg) is used as a positive control. **A** Diagram illustrating the mouse model of colitis employed in this study. **B** Percentage body weight change (*n* = 6). **C** DAI scores (*n* = 6). **D**, **E** Colon length (*n* = 6). **F**, **G** H&E staining of colon (*n* = 3). **H** Expression level of inflammation related factors in colon tissue (*n* = 6). Data are expressed as the means ± S.E.M., ^##^*P* < 0.01 *versus* Normal, *P < 0.05 and **P < 0.01 *versus* Model

To investigate the therapeutic effect of isoimperatorin in UC, DSS-induced colitis mice were administered isoimperatorin, using 5-ASA as a positive control (Fig. 1A). Compared to the normal group, model group mice exhibited progressive weight loss and colitis-associated symptoms, including diarrhea, bloody stool, and perianal soiling. Oral gavage of isoimperatorin (20 and 40 mg/kg) mitigated weight loss (Fig. 1B) and reduced disease activity index (DAI) scores (Fig. 1B, C). In addition, colon shortening, a hallmark of DSS-induced colitis, was observed in model mice compared to normal. Isoimperatorin treatment significantly inhibited the shortening of the length of the colon (Fig. 1D, E). Similarly, isoimperatorin treatment remarkably ameliorated colonic histopathological damage and reduced pathological scores in colitis mice (Fig. 1F, G). What’s more, TNF-α, IL-6, and IL-1β mRNA levels were significantly elevated in the colonic tissues of mice in the model group compared with the normal group, and isoimperatorin treatment significantly suppressed the expression of pro-inflammatory factors (Fig. 1H). Altogether, these findings demonstrate that intervention with isoimperatorin improves symptoms in UC mice.

### Isoimperatorin induces Treg cell generation in MLNs of DSS-induced mice

**Fig. 2:**
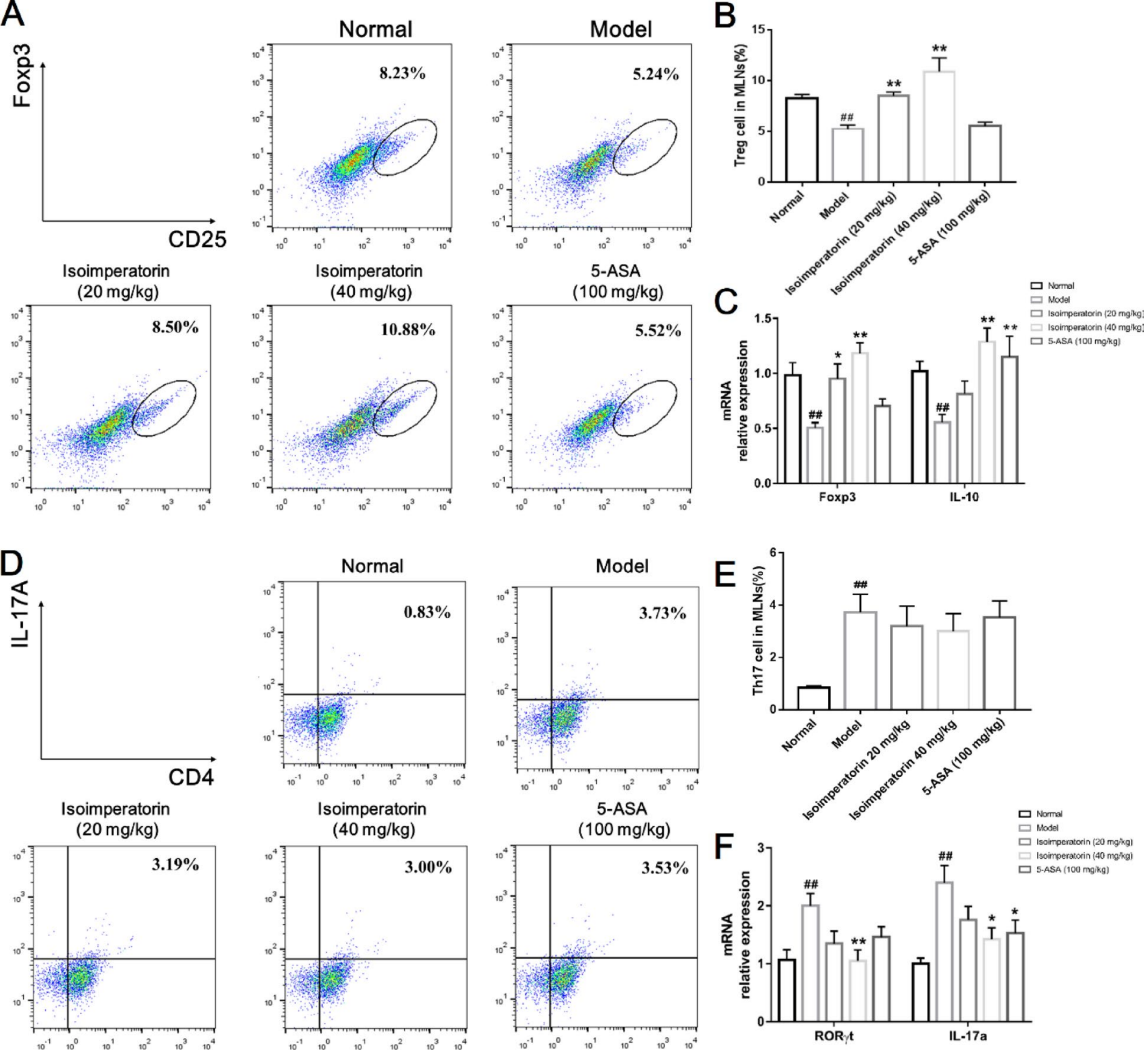
Isoimperatorin mainly induces Treg cell generation in MLNs of DSS-induced mice. Isoimperatorin (20, 40 mg/kg) was orally administered for consecutive 7 days. 5-ASA (100 mg/kg) is used as a positive control. **A**, **B** The frequency of Treg cells in the MLNs (*n* = 3). **C** The mRNA expression levels of Foxp3 and IL-10 in the MLNs (*n* = 6). **D**, **E** The frequency of Th17 cells in the MLNs (*n* = 3). **F** The mRNA expression levels of RORγt and IL-17a in the MLNs (*n* = 6). Data are expressed as the means ± S.E.M., ^##^*P* < 0.01 *versus* Normal, *P < 0.05 and **P < 0.01 *versus* Model

Because of the potential immunomodulatory effects of isoimperatorin, its impacts on Treg cell generation were first investigated. The results demonstrated that the proportion of Treg cells in the MLNs of model group mice was significantly decreased compared to normal group. Oral administration of isoimperatorin significantly elevated the proportion of Treg cells in the MLNs (Fig. 2A, B). Similarly, the expression levels of Foxp3 and IL-10 mRNA in MLNs were significantly reduced in the model group, and the expression levels of Foxp3 and IL-10 mRNA were significantly up-regulated after treatment with isoimperatorin (Fig. 2C). Subsequently, the role of isoimperatorin on Th17 cell proportion was examined. The results showed that compared to normal group, the proportion of Th17 cells in the MLNs of model group mice was significantly increased. While oral administration of isoimperatorin only slightly inhibit the proportion of Th17 cells in MLNs (Fig. 2D, E). Similarly, the expression levels of ROR γt and IL-17a mRNA in MLNs of the model group mice were significantly increased. Only high-dose isoimperatorin effectively suppressed these inflammatory markers (Fig. 2F). The above results suggest that isoimperatorin primarily induces the generation of Treg cells and exerts immunomodulatory effects.

### Isoimperatorin promotes intestinal mucosal healing and restores barrier function

**Fig. 3:**
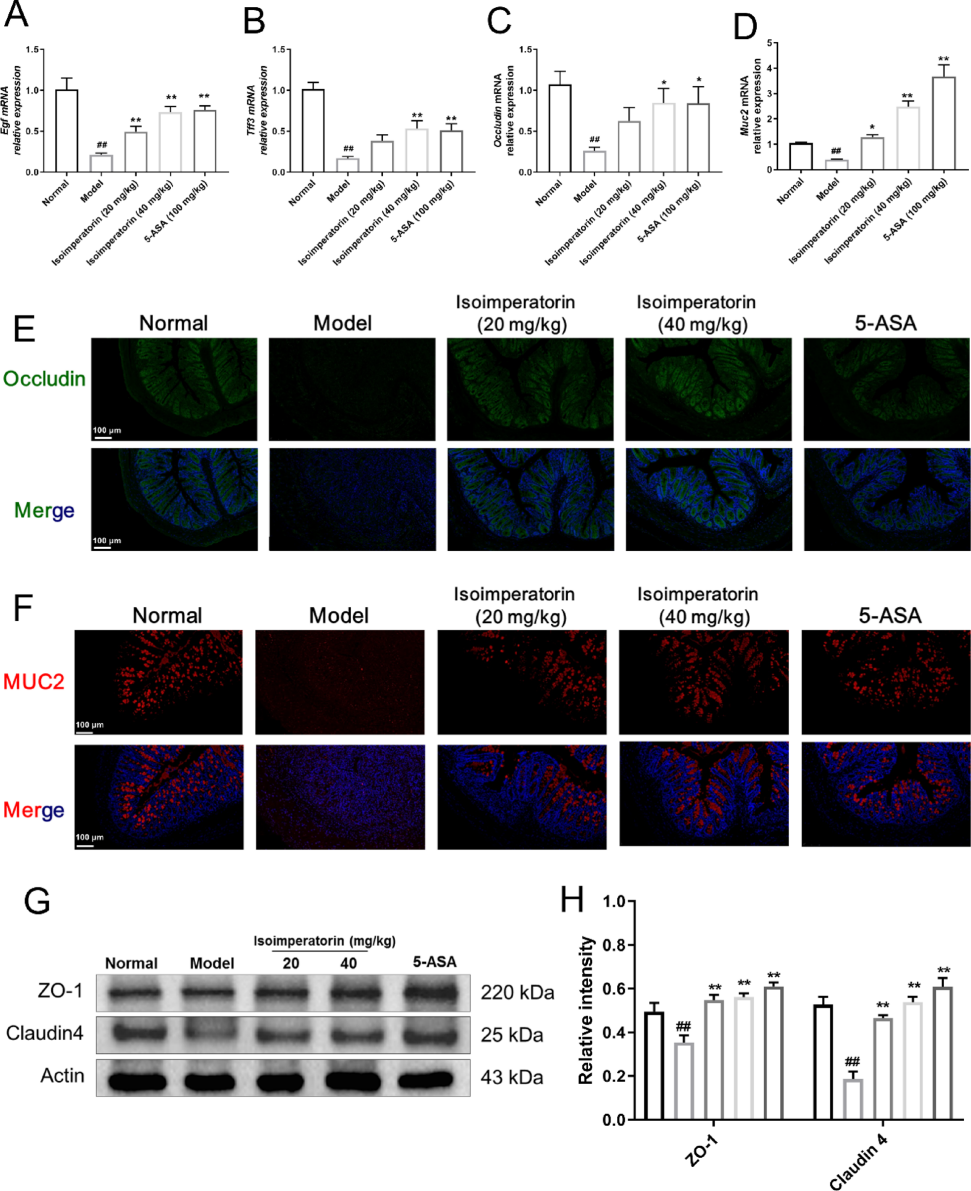
Isoimperatorin promotes intestinal mucosal healing and restores barrier function. Isoimperatorin (20, 40 mg/kg) was orally administered for consecutive 7 days. 5-ASA (100 mg/kg) is used as a positive control. **A**–**D** The mRNA expression levels of EGF, TFF3, Occludin and MUC2 in the colon tissue (*n* = 6). **E**, **F** Immunofluorescence staining for Occludin and MUC2 in colon tissue (*n* = 3). **G**, **H** The expression levels of ZO-1 and Claudin4 protein in colon tissue (*n* = 3). Data are expressed as the means ± S.E.M., ^##^*P* < 0.01 versus Normal, *P < 0.05 and **P < 0.01 *versus* Model

Mucosal healing has emerged as a key therapeutic goal in UC treatment (Neurath and Vieth 2023). Therefore, the ability of isoimperatorin to promote intestinal mucosal healing and repair the damaged intestinal barrier in UC mice was evaluated. The results showed that compared to normal group, the expression of mucosal healing genes (EGF, TFF3) was significantly reduced in the model group mice, while treatment with isoimperatorin could upregulated the expression levels of mucosal healing-related factors (Fig. 3A, B). Similarly, the expression levels of intestinal epithelial barrier functional proteins (MUC2, Occludin) and integrity proteins (ZO-1 and Claudin4) were significantly reduced in the model group. The intervention of isoimperatorin increased the expression levels of functional and integrity proteins in the intestinal epithelial barrier (Fig. 3C–H). The above research results demonstrate that isoimperatorin can promote intestinal mucosal healing and restore barrier function in UC mice.

### Isoimperatorin accelerates the migration of colonic epithelial cells and promotes wound healing

**Fig. 4:**
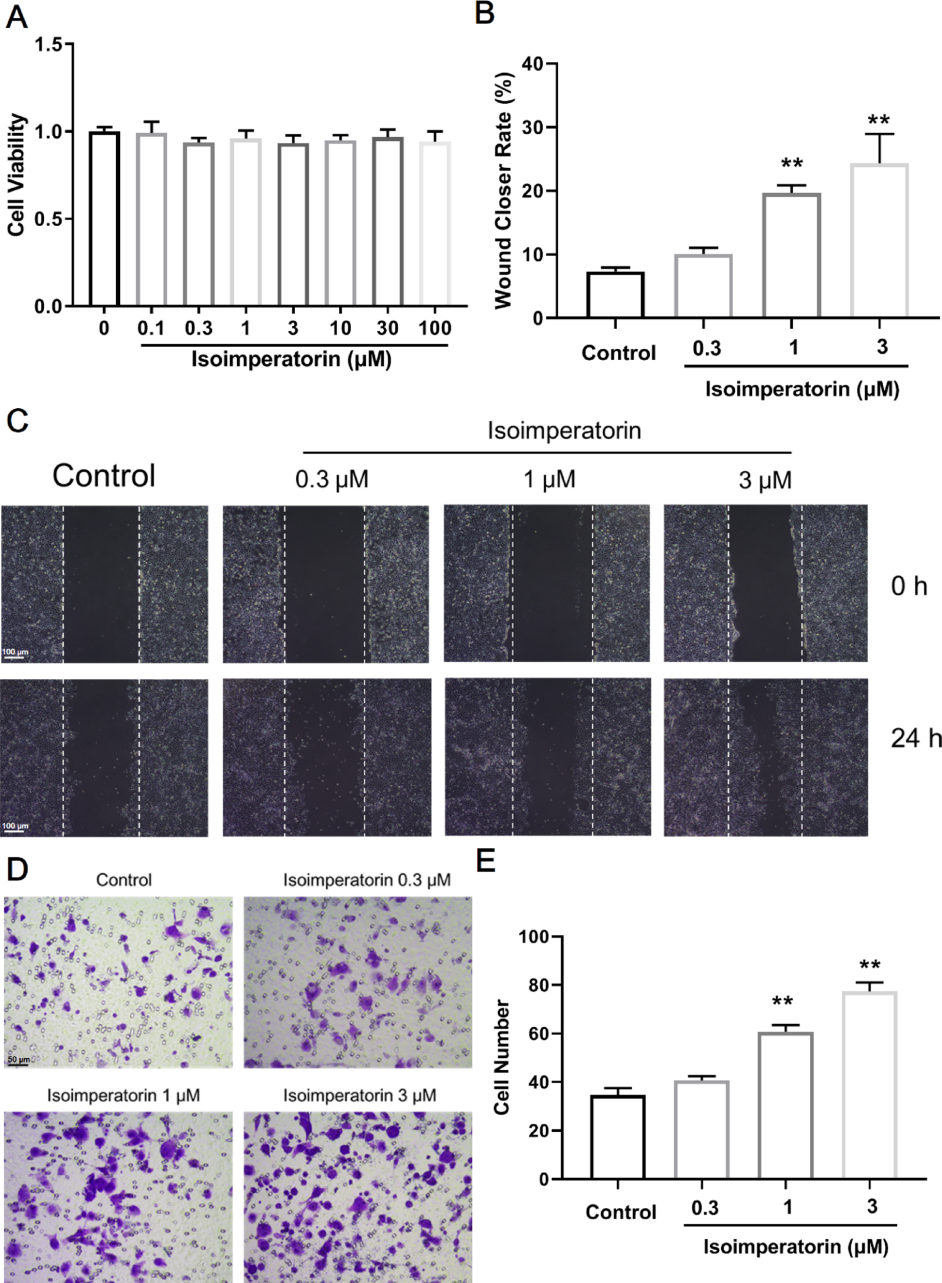
Isoimperatorin accelerates the migration of colonic epithelial cells and promotes wound healing. NCM460 cells were cultured in RPMI 1640 medium, maintained at 37 °C with 5% CO_2_. **A** Examination of the effect of isoimperatorin on NCM 460 cell viability using the CCK-8 assay (*n* = 6). **B**, **C** Cell scratch assay to examine the effect of isoimperatorin on wound healing in NCM460 cells (*n* = 3). **D**, **E** Tanswell assay to examine the impact of isoimperatorin on NCM460 cell migration (*n* = 3). Data are expressed as the means ± S.E.M., *P < 0.05 and **P < 0.01 *versus* Control

The epithelial cells near the site of intestinal mucosal injury migrate to the wound and re-seal the exposed basement membrane. The closer to the wound site, the faster the migration speed of epithelial cells (Sturm and Dignass 2008). Firstly, the CCK-8 assays were used to assessed the effect of isoimperatorin on the viability of NCM 460 cells. The results showed that isoimperatorin (0.1, 0.3, 1, 3, 10, 30, 100 µM) had no significant effect on the viability of NCM 460 cells (Fig. 4A). The scratch assays were used to investigate the effect of isoimperatorin on wound healing in NCM460 cells, and the results revealed that isoimperatorin (1, 3 µM) significantly promoted wound healing in NCM460 cells (Fig. 4B, C). Transwell migration assays were used to investigate the effect of isoimperatorin on NCM460 cell migration, and the results demonstrated that isoimperatorin (1, 3 µM) promoted the migration of NCM460 cells (Fig. 4D, E). The above research results indicate that isoimperatorin accelerates the migration of colonic epithelial cells and promotes wound healing.

**Fig. 5:**
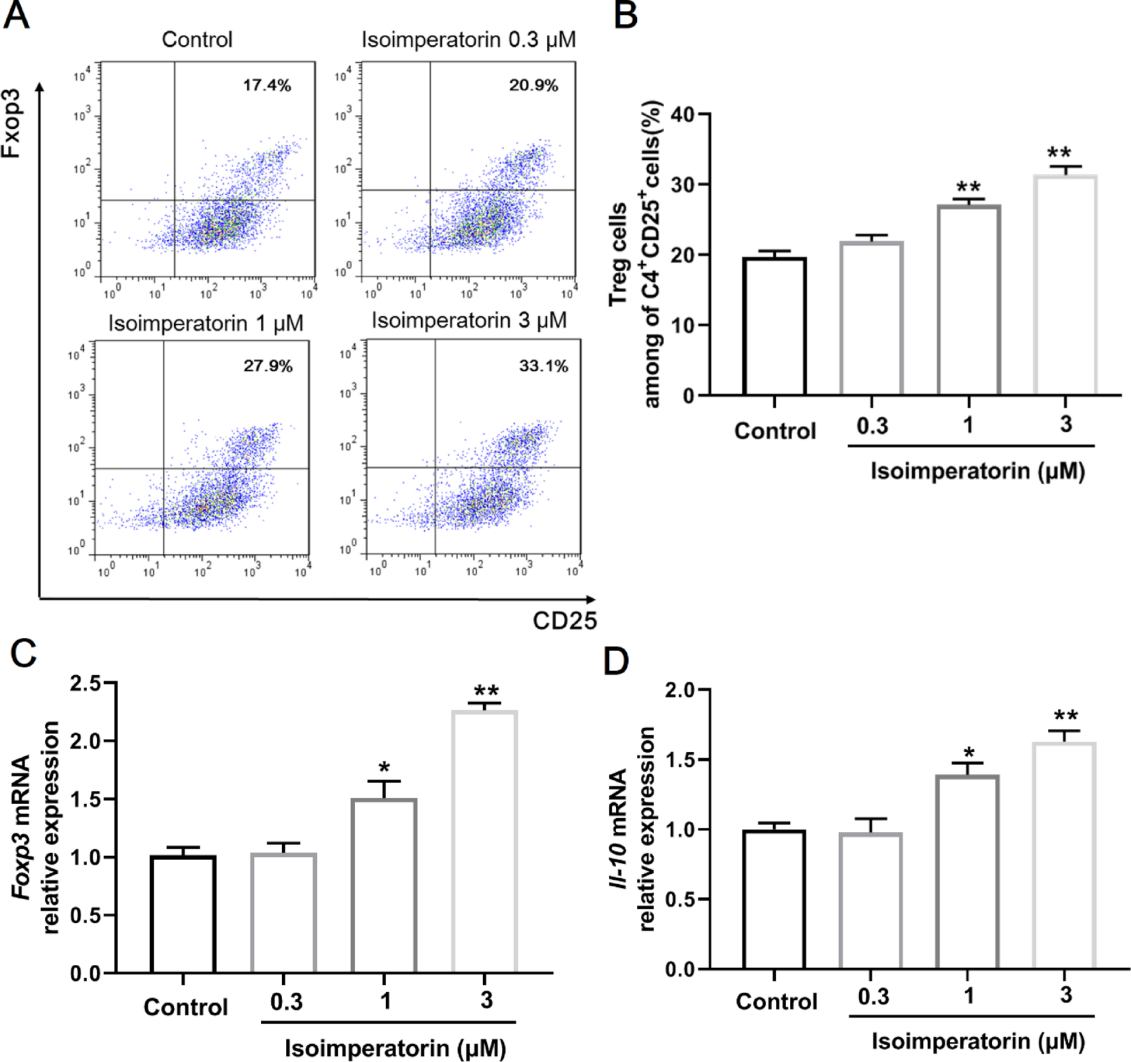
Isoimperatorin induces Treg cell generation in vitro. Naïve CD4 + T cells were cultured with CD3 (3 µg/mL) and CD28 (2 µg/mL) antibodies. TGF-β (3 ng/mL) and IL-2 (100 U/mL) were added to induce Treg cell differentiation. Cells were cultured for 72 h in the presence or absence of isoimperatorin (0.3, 1, 3 µM). **A**, **B** Frequencies of Treg cells were analysed by flow cytometry (*n* = 3). **C**, **D** The mRNA expression of Foxp3 and IL-10 was analysed by Q-PCR (*n* = 3). Data are expressed as the means ± S.E.M., *P < 0.05 and **P < 0.01 *versus* Control

### Isoimperatorin induces Treg cell generation in vitro

At the same time, naïve CD4+ T cells can differentiate into Treg cells under conditions of activation stimulation (Fantini et al. 2007). Thus, the role of isoimperatorin in inducing Treg cell generation was examined in vitro. The results showed that under the conditions of Treg cell generation, isoimperatorin (1, 3 µM) significantly increased the percentage of Treg cells compared with the control (Fig. 5A, B). In addition, isoimperatorin treatment upregulated the mRNA expression of Foxp3 and IL-10 compared with the control under conditions of Treg cell generation in vitro (Fig. 5C, D). The results of these experiments demonstrate that isoimperatorin can significantly induce Treg cell generation in vitro.

### Depletion of Treg cell weakens the anti-colitis effect of isoimperatorin

**Fig. 6:**
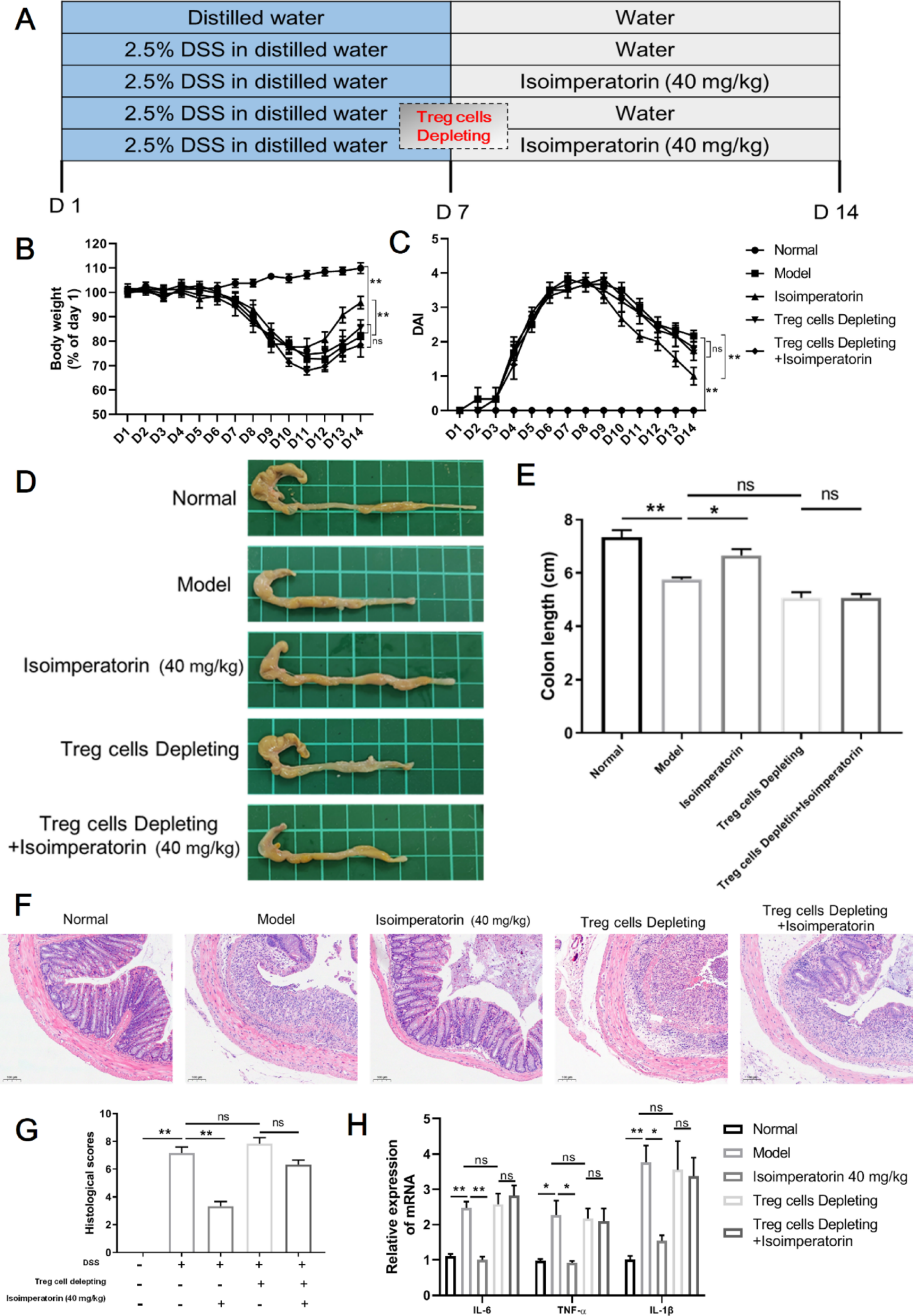
Depletion of Treg cell weakens the anti-colitis effect of isoimperatorin. Colitis was induced in male C57BL/6 mice by drinking with 2.5% DSS for 7 days, followed by normal drinking water for 7 days. Treg cells were depleted via tail vein injection of anti-CD25 antibody (200 µg/mouse) on day 7. Isoimperatorin (40 mg/kg) was orally administered for consecutive 7 days. **A** Diagram illustrating the mouse model of colitis employed in this study. **B** Percentage body weight change (*n* = 6). **C** DAI scores (*n* = 6). **D**, **E** Colon length (*n* = 6). **F**, **G** H&E staining of colon (*n* = 3). **H** The expression level of inflammation related factor in colon tissue (*n* = 6). Data are expressed as the means ± S.E.M. *P < 0.05 and **P < 0.01 *versus* comparable groups

To further clarify whether isoimperatorin exerts anti-colitis activities by inducing Treg cell generation, purified anti-CD25 mouse antibody was injected *via* tail vein to deplete Treg cells in vivo, and the effect of isoimperatorin on ameliorating UC was assessed (Fig. 6A). The results showed that oral gavage isoimperatorin (40 mg/kg) restored body weight loss, decreased DAI score, and inhibited shortening of colon length in UC mice. After the depletion of Treg cells, the effects of isoimperatorin on restoring weight loss, decreasing DAI score, and inhibiting shortening of colon length were significantly impaired (Fig. 6B–D). Similarly, isoimperatorin significantly ameliorated colonic histopathological damage and inhibited TNF-α, IL-6, and IL-1β mRNA expression in UC mice. After depletion of Treg cells, the effects of isoimperatorin on ameliorating the pathological damage of colon tissues and inhibiting the expression of inflammatory factors were significantly attenuated (Fig. 6F–H). The above findings suggest that depletion of Treg cells significantly inhibited the anti- colitis effects of isoimperatorin.

### Depletion of Treg cell counteracts the effects of isoimperatorin in promoting Treg cell generation

**Fig. 7:**
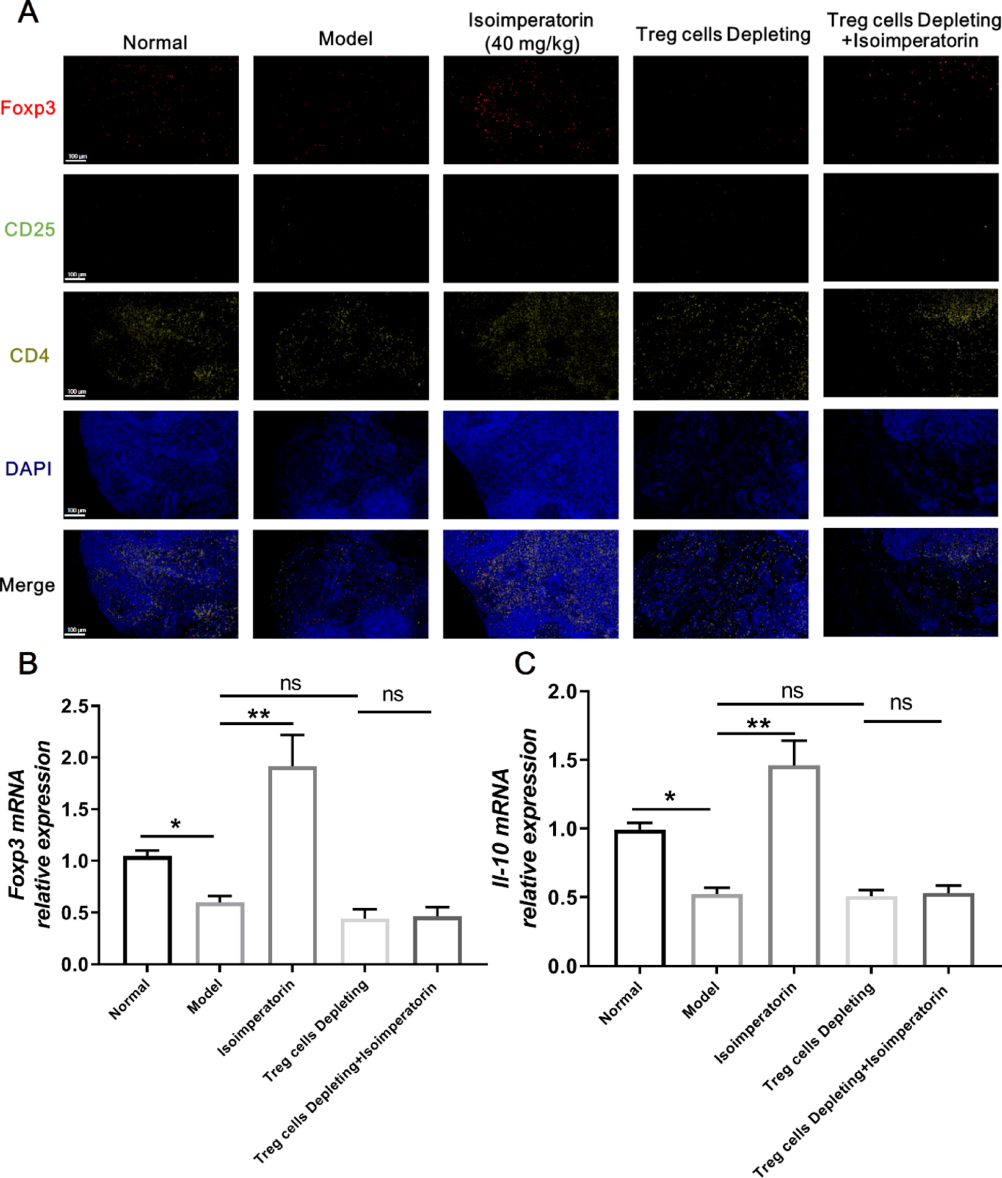
Depletion of Treg cell counteracts the effects of isoimperatorin in promoting Treg cell generation. Purified anti-CD25 (200 µg/mouse) was injected into the tail vein on day 7 to deplete Treg cells in mice. Isoimperatorin (40 mg/kg) was orally administered for consecutive 7 days. **A** Immunofluorescence staining for CD4 + CD25 + Foxp3 + T in MLNs (*n* = 3). **B**, **C** The mRNA expression levels of Foxp3 and IL-10 in the MLNs (*n* = 6). Data are expressed as the means ± S.E.M., *P < 0.05 and **P < 0.01 *versus* comparable groups

Subsequently, Treg cells were identified as CD4^+^CD25^+^Foxp3^+^ T cells in MLN tissues. Immunofluorescence was employed to assess the effect of isoimperatorin-induced Treg cell generation after the depletion of Treg cells. The results showed that isoimperatorin treatment significantly increased the number of Treg cells in MLN tissues. Following Treg cell depletion, isoimperatorin-induced Treg cell generation was markedly attenuated in MLN tissues (Fig. 7A). In addition, isoimperatorin treatment enhanced the expression of Foxp3 and IL-10 in MLN tissues. As Treg cells were depleted, the above effects of isoimperatorin were significantly diminished (Fig. 7B, C). These data further confirm the concept that isoimperatorin induces Treg cell generation and thus exerts an anti-colitis effect.

### Depletion of Treg cell impairs the effects of isoimperatorin in promoting intestinal mucosal healing and restoring barrier function

**Fig. 8:**
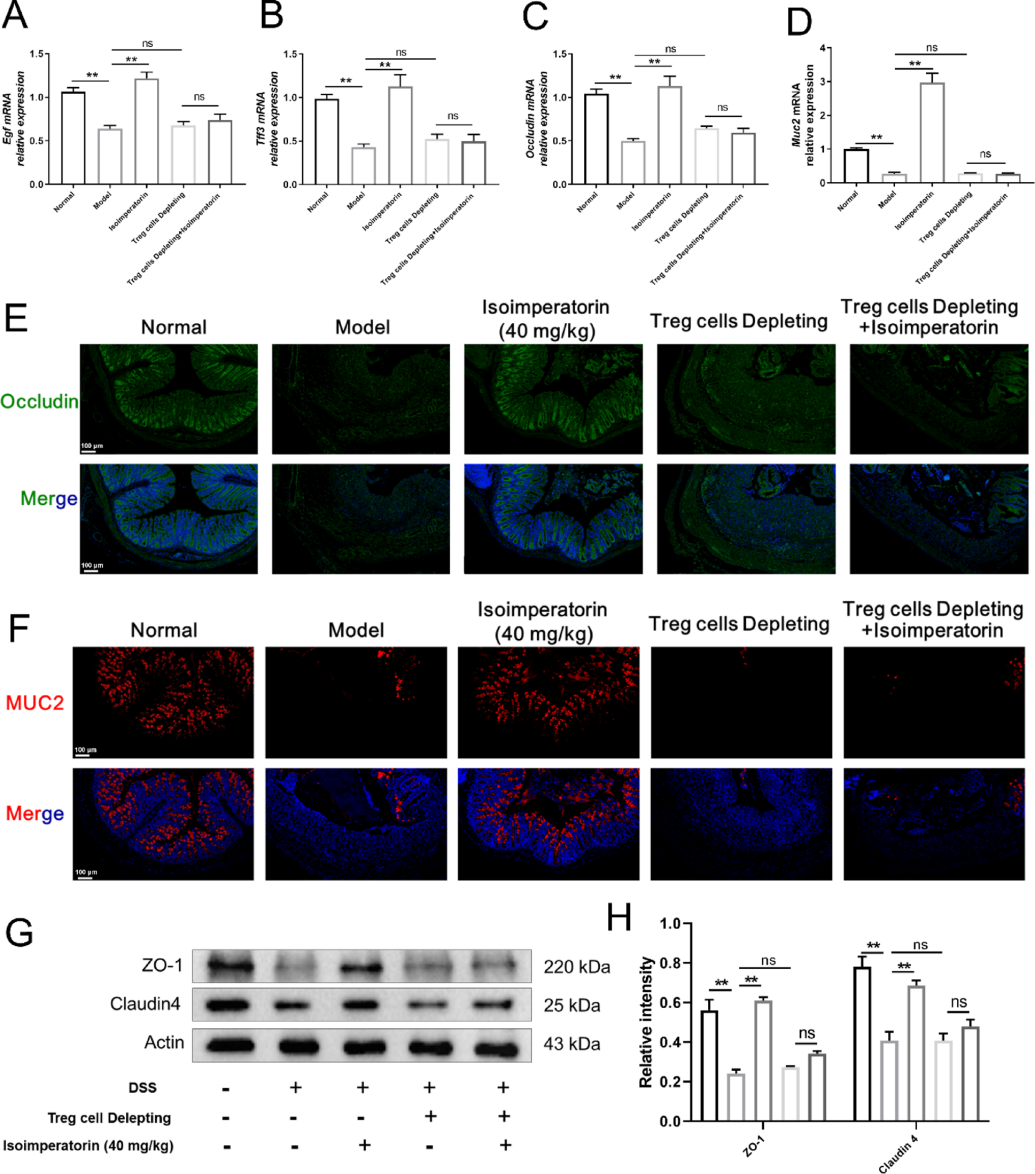
Depletion of Treg cell impairs the effects of isoimperatorin in promoting intestinal mucosal healing and restoring barrier function. Purified anti-CD25 (200 µg/mouse) was injected into the tail vein on day 7 to deplete Treg cells in mice. Isoimperatorin (40 mg/kg) was orally administered for consecutive 7 days. **A**–**D** The mRNA expression levels of EGF, TFF3 and Occludin in the colon tissue (*n* = 6). **E**, **F** Immunofluorescence staining for Occludin and Muc2 in colon tissue (*n* = 3). **G**, **H** The expression levels of ZO-1 and Claudin4 protein in colon tissue (*n* = 3). Data are expressed as the means ± S.E.M., *P < 0.05 and **P < 0.01 *versus* comparable groups

Consistent with the earlier findings, isoimperatorin intervention was able to elevate the expression levels of mucosal healing-associated factors (EGF, TFF3), and isoimperatorin pro-mucosal healing activity was abolished following Treg cell depletion (Fig. 8A, B). In addition, isoimperatorin treatment upregulated the expression levels of intestinal epithelial barrier functional (Occludin and MUC2) and integrity (ZO-1 and Claudin4) proteins. Upon depletion of Treg cells, the above effects of isoimperatorin were significantly attenuated (Fig. 8C–H). The above findings demonstrate that the effects of isoimperatorin on promoting intestinal mucosal healing and restoring barrier function are dependent on Treg cell generation.

## Discussion

Isoimperatorin is a natural furanocoumarin compound mainly derived from Umbelliferae plants (with Baizhi being the most representative). Modern pharmacological studies demonstrate that isoimperatorin exhibits diverse pharmacological activities such as anti-inflammation and analgesia, prevention of osteoporosis, neuroprotection, inhibition of angiogenesis, and anti-tumor (Fan et al. 2023; Li et al. 2023; Xu et al. 2024; Rajendran et al. 2023; Kim et al. 2022). In the present study, a mouse model of DSS-induced colitis was employed to explore the anti-UC effects of isoimperatorin based on an immunomodulatory perspective. Isoimperatorin was able to restore body weight, reduce DAI score, inhibit shortening of colon length, reduce the histopathological score, and significantly improve UC disease symptoms in model mice. Moreover, the anti-colitis effect of isoimperatorin showed significant correlation with Treg cell generation. While our findings demonstrate consistent trends, we recognize that certain experiments utilized limited biological replicates due to technical constraints in parallel animal model preparation. Future studies with larger cohorts will strengthen statistical power and generalizability. Notwithstanding these considerations, the current work systematically establishes a conceptual framework that supports further exploration of isoimperatorin’s clinical potential, particularly in therapeutic development.

Disorders of the immune system are recognized as the primary driver of intestinal inflammation in UC, which is significantly characterized by the disruption of immune tolerance mediated through Treg cells. Studies have shown that the proportion of Treg cells in the peripheral blood and the lamina propria of the colonic mucosa are significantly reduced in UC patients (Ueno et al. 2013). Notably, refractory UC patients receiving Treg cell transfer therapy showed significant clinical improvement with reduced disease activity scores (Voskens et al. 2023). As immunosuppressive cells, Treg cells exert negative regulatory effects in UC pathogenesis, highlighting their therapeutic potential as treatment targets (Neurath 2024). In the present study, isoimperatorin was able to induce Treg cell generation and promote mRNA expression of Foxp3 and IL-10 both in vivo and in vitro. Importantly, Treg cell depletion markedly attenuated isoimperatorin’s anti-colitis effects, confirming its Treg-dependent mechanism. Classically, TGF-β binds to its receptor and activates Smad2/3 proteins to form a complex. This complex translocates into the nucleus, directly binds to the Foxp3 gene promoter, induces Foxp3 expression, and drives the differentiation of naive T cells into Treg cells (Wang et al. 2023). Similarly, IL-2 signaling involves ligand-receptor binding and JAK kinase activation, leading to STAT5 phosphorylation. The phosphorylated STAT5 dimerizes and translocates into the nucleus, where it directly binds to the enhancer region of the Foxp3 gene to promote its transcription (Zong et al. 2024). Despite these well-characterized pathways, the precise mechanisms underlying isoimperatorin-induced Treg cell generation require further investigation.

Mucosal healing has emerged as a key therapeutic target in UC treatment guidelines. However, there are no drugs that directly promote colonic mucosal healing in clinical application (Turner et al. 2021). TCM and its active ingredients have the advantages of wide adaptability, fewer side effects, and safe and reliable effects in the treatment of UC. Therefore, finding anti-UC drugs from TCM that directly facilitate mucosal healing has great clinical application value (Wang et al. 2024). In the present study, isoimperatorin enhanced the expression of mucosal healing-related factors while improving intestinal epithelial barrier integrity through upregulation of functional proteins, ultimately promoting mucosal repair and restoring barrier function in mice with colitis. While this study primarily focuses on elucidating the therapeutic potential of isoimperatorin in acute colitis, the translational implications of this intervention in chronic disease phases remain to be explored. Importantly, extending these investigations to chronic colitis models could yield critical insights into mucosal repair dynamics, thereby informing clinical strategies for sustained epithelial homeostasis. Collectively, the findings establish a foundation that accelerates the development of next-generation mucosal repair therapeutics, with isoimperatorin serving as a promising lead compound.

The process of intestinal mucosal healing involves complex interactions between immune cells and epithelial cells (Villablanca et al. 2022). During the initial healing phases, numerous immune cells (such as neutrophils and macrophages) accumulate, triggering an inflammatory response and creating a microenvironment favorable for epithelial regeneration and mucosal healing (Brazil et al. 2019). Within hours or days after mucosal injury, epithelial cells begin to proliferate, followed by epithelial cell motility, with epithelial cells near the wound migrating to the injury site to reseal the exposed basement membrane (Iizuka and Konno 2011). In the present study, CCK-8 assays demonstrated that isoimperatorin had no significant effect on epithelial cell proliferation. It was noteworthy that isoimperatorin significantly accelerated epithelial cell migration and thus promoted wound healing. While these findings improve our understanding of the disease mechanisms, they lack validation in human-relevant models-particularly from primary intestinal epithelial cells or clinical biopsy tissues from UC patients. We believe that future studies should incorporate patient-derived organoid tissues with systematic preclinical validation to determine the pharmacodynamic heterogeneity of isoimperatorin.

## Conclusion

**Fig. 9:**
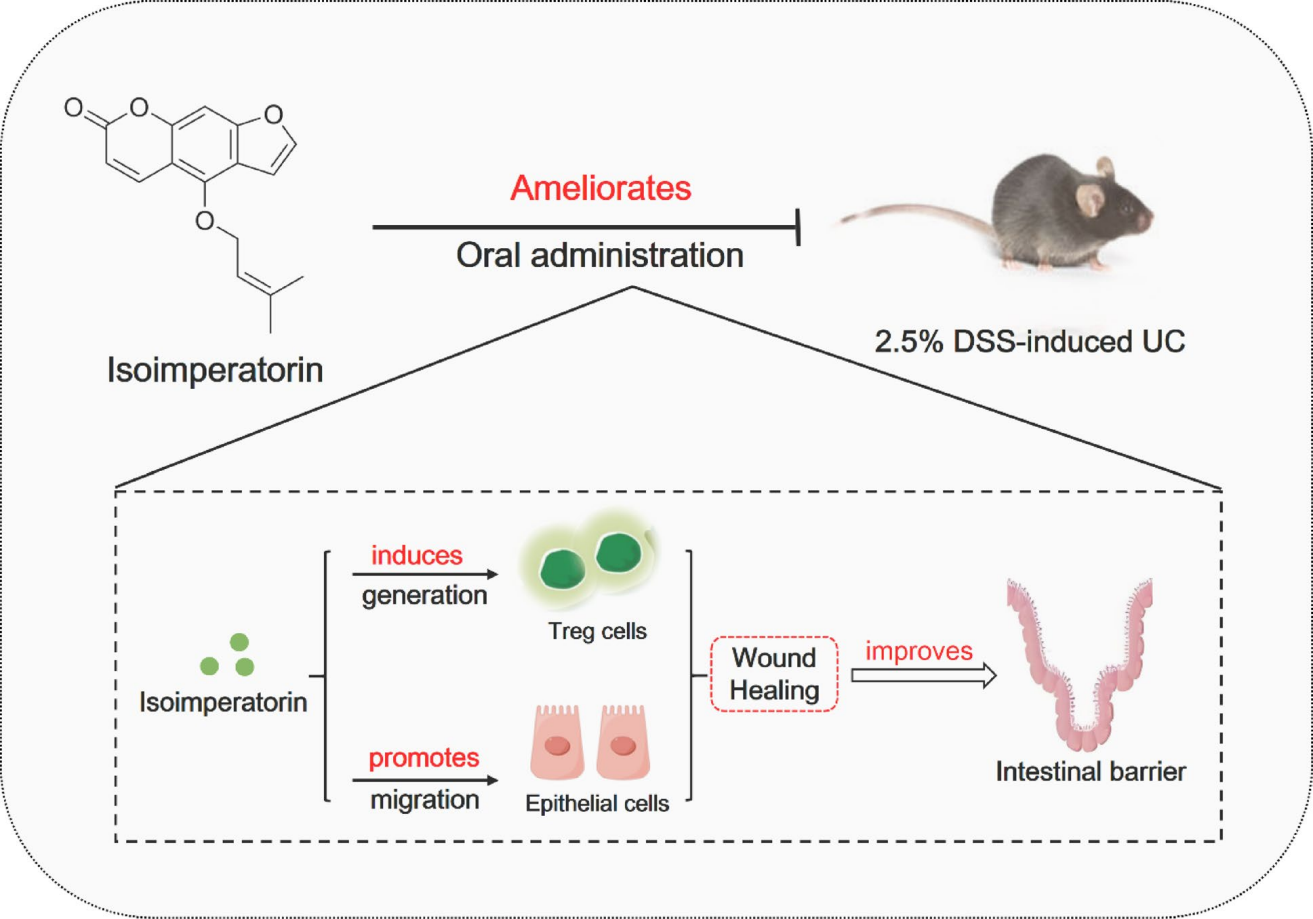
Isoimperatorin ameliorates ulcerative colitis by inducing Treg cell generation and promoting mucosal healing

In summary, isoimperatorin significantly alleviated DSS-induced colitis symptoms, induced Treg cell generation in MLNs, promoted intestinal mucosal healing, and restored barrier function (Fig. 9). It is worth noting that the improvement of colitis by isoimperatorin showed a Treg cell-dependent effect. The present study provides a theoretical basis for the clinical application and development of isoimperatorin.

## Data Availability

The datasets used and/or analysed during the current study are available from the corresponding author on reasonable request.

## Abbreviations

5-ASA: 5-aminosalicylic acid
DAI: Disease activity index
DSS: Dextran sulfate sodium
MLN: Mesenteric lymph node
TCM: Traditional Chinese medicine
UC: Ulcerative colitis
